# Lactate drives CD38 signaling to promote Epithelial‐Mesenchymal Transition through Snail induction in non‐small cell lung cancer cells

**DOI:** 10.1002/ccs3.12018

**Published:** 2024-02-14

**Authors:** Yating Lu, Yang Yang, Tao Chang, Qiyun Jiang, Chenfeng Yang, Chunzhe Fu, Huijun Wei, Yuanpeng He, Zhihao Wu

**Affiliations:** ^1^ Research Laboratory of Tumor Microenvironment Wannan Medical College Wuhu China; ^2^ School of Clinical Medicine Wannan Medical College Wuhu China; ^3^ School of Public Health Wannan Medical College Wuhu China; ^4^ School of Pharmacy Wannan Medical College Wuhu China; ^5^ Provincial Engineering Laboratory for Screening and Re‐evaluation of Active Compounds of Herbal Medicines in Southern Anhui Wannan Medical College Wuhu China; ^6^ School of Anesthesiology Wannan Medical College Wuhu China; ^7^ Anhui Province Key Laboratory of Active Biological Macro‐molecules Research Wannan Medical College Wuhu China; ^8^ Key Laboratory of Non‐coding RNA Transformation Research of Anhui Higher Education Institution Wannan Medical College Wuhu China; ^9^ Anhui Provincial Engineering Research Center for Dental Materials and Application Wannan Medical College Wuhu China

**Keywords:** CD38, EMT, lactate, lung cancer, Snail, TAZ

## Abstract

CD38 is the main NADase in mammalian cells. It regulates the homeostasis of nicotinamide adenine dinucleotide (NAD+) and extracellular nucleotides. Its function plays an important role in infection and aging. However, its potential functions in tumor cells have not been fully elucidated. In the present study, we demonstrated that lactate, which is derived from tumor metabolism remodeling, upregulates the expression of CD38 through OXPHOS‐driven Hippo‐TAZ pathway. The highly expressed CD38 converts NAD + to adenosine through the CD203a/CD73 complex and adenosine binds and activates its receptor A2AR, inducing the expression of Snail and promoting the invasion and metastasis of lung cancer cells. This finding elucidates a new perspective on the interplay between NAD + metabolism and glycolysis in tumor development.

## Graphical abstract

Lactic acid derived from tumor metabolic reprogramming upregulates the expression of CD38 through the OXPHOS‐driven Hippo‐TAZ pathway, promoting the conversion of nicotinamide adenine dinucleotide (NAD+) to adenosine and activating adenosine receptors. The activated adenosine receptors induce the expression of Snail through the Akt‐Gsk3β pathway, promoting the progression of EMT in lung cancer cells.

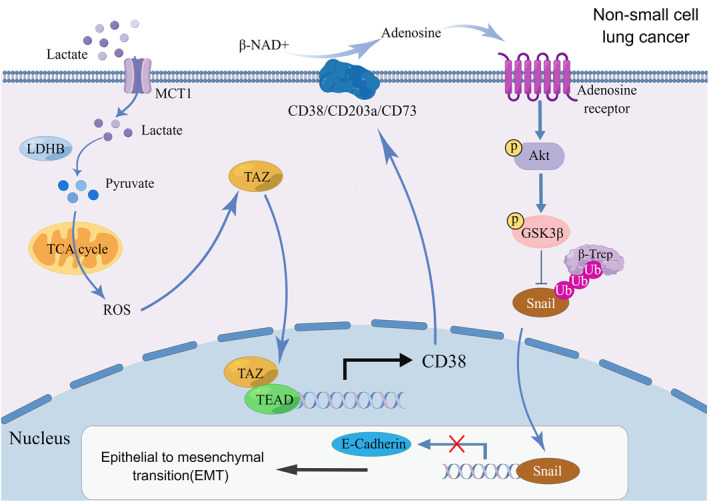

## 1 INTRODUCTION

Metastasis is a malignant sign and characteristic of lung cancer and it is also the primary cause of treatment failure and death of lung cancer patient. The majority of lung cancer patients already have local or distant metastasis at the initial diagnosis and thus a poor prognosis;^1^ therefore, a better understanding of lung cancer metastasis is essential for developing effective therapeutic strategies for lung cancer.

The invasion and metastasis of tumors are complex processes that involve multiple genes and are completed in multiple steps. However, the detachment of tumor cells from the primary lesion and infiltration into the surrounding stroma are considered the key steps of tumor invasion and early metastasis. Epithelial–Mesenchymal Transition (EMT) is a fundamental physiological phenomenon that occurs in the early stages of embryonic development, characterized by the loss of epithelial cell phenotype and acquisition of migratory mesenchymal cell characteristics. Malignant epithelial tumor cells activate the dormant EMT process to break free from the tight connections between epithelial cells, acquiring invasiveness to infiltrate surrounding tissues and spread to distant organs.^2^ EMT is a strictly regulated process mediated by several transcriptional factor such as Snail and Twist. It has been demonstrated that overexpression of Snail induce resistance to cell apoptosis and chemotherapy drugs in addition to promoting EMT, indicating that Snail‐induced EMT program plays a critical role in tumor progression.^3^

CD38 is a type II single‐chain type II transmembrane glycoprotein located on the cell surface, which has multiple functions. It is both an enzyme protein and a cell surface receptor, and it plays an important role in the process of immune regulation. The enzymatic activity of CD38 is manifested by catalyzing the conversion of nicotinamide adenine dinucleotide (NAD+) into metabolites such as ADP‐ribose (ADPR) and cyclic ADP‐ribose (cADPR). CD38 is the major NADase in mammalian cells and determines the intracellular levels of NAD.^4^ Studies have shown that knocking out CD38 in mice significantly increases the concentration of NAD and the SIRT deacetylase activity (Camacho‐Pereira et al., 2016). CD38 increases with age and may lead to age‐related NAD decline.^5^ In addition, CD38 is aberrantly expressed in various types of tumors including lung cancer.^6^, ^7^, ^8^ However, the mechanism behind the increase in CD38 expression in tumor cells is not yet clear.

The products of CD38 catalytic reactions, such as ADPR and cADPR, act as important second messengers in cells, which can affect cell growth, insulin release, T cell activation and other functions by regulating intracellular calcium ions.^4^ The ADPR/cADPR generated by CD38 can be further catalyzed by enzymes such as CD203 and CD73 to produce adenosine, providing a secondary pathway for the production of extracellular adenosine bypassing CD39.^9^ Taken together, the multiple functions of CD38 in the microenvironment ultimately decrease extracellular NAD+, alter calcium signaling cascades, and produce immunosuppressive adenosine. The A2A receptor (A2AR), a receptor with high affinity for adenosine, is a typical GPCR expressed in many immune cells.^10^ Adenosine binds to the A2AR receptor, activates the typical *G* protein, and triggers the cAMP/PKA/CREB signaling pathway. The activation of A2AR signaling pathway inhibits the immune response of immune cells, thereby promoting immune escape of tumor cells in the tumor microenvironment.^11^ However, the expression of A2AR is significantly increased in many solid tumors, and in gastric cancer, the adenosine‐A2AR signaling pathway promotes tumor metastasis.^12^ However, it is not clear whether the CD38‐adenosine‐A2AR signaling pathway is involved in lung cancer invasion and progression, and its potential molecular mechanism is not very clear.

Metabolic reprogramming causes tumor cells to rely primarily on aerobic glycolysis for energy production (Warburg effect), leading to a significant accumulation of lactate in the tumor microenvironment. While traditionally considered a “metabolic waste product” of tumor cell glycolysis, lactate has recently been demonstrated to play an important role in tumor immune evasion, evasion of senescence in pre‐cancerous cells, as well as tumor resistance to drugs and metastasis.^13^, ^14^, ^15^, ^16^ In the present study, we demonstrated that lactate‐driven oxidative phosphorylation (OXPHOS) significantly increased CD38 expression by activating the Hippo pathway. The CD38‐adenosine‐A2AR axis, in turn, regulates Snail‐dependent lung cancer cell EMT. Our data indicate that CD38 plays a critical role in lung cancer development and that anti‐CD38 may have therapeutic potential in lung cancer.

## 2 MATERIALS AND METHODS

### 2.1 Cells, transfection, antibodies, plasmids, and reagents

The A549 (human lung adenocarcinoma), H1299 (human lung adenocarcinoma) cell lines were cultured with DMEM (Hyclone, Logan, UT, USA) containing 10% fetal bovine serum (FBS, Gibco BRL, Grand Island, NY, USA) at 37°C in a humidified atmosphere of 5% CO_2_. For transfection, A549 and H1299 cells were seeded in six‐well plates (10^5 cells per well) and were grown to 70%–90% confluence before plasmids transfection with PolyJet DNA Transfection Reagent (SignaGen Laboratories, Gaithersburg, MD, USA), according to the manufacturer's instructions. Anti‐CD38 (#51000), anti‐AKT (#9272), anti‐p‐AKT (#4060), anti‐N‐cadherin (#4061), anti‐TAZ (#8418), anti‐p‐CREB (#9198), anti‐CREB (#9197), anti‐TEAD1 (#12292), and anti‐Snail (#3879) antibodies were purchased from Cell Signaling Technology (Danvers, MA, USA). Anti‐β‐actin (#A1978) and anti‐E‐cadherin (SAB4503751) antibodies were obtained from Sigma (Victoria, BC, Canada). Anti‐CD73 (ab133582) and anti‐Fibronectin (ab299) were purchased from Abcam (Cambridge, UK). Anti‐Fibronectin (A0850) antibodies was obtained from ABclonal (ABclonal, China). The HA‐TAZ, HA‐TEAD1, HA‐CD38 were purchased from Addgene (Cambridge, MA, USA). Regadenoson (HY‐A0168), adenosine A2A receptor inhibitor (HY‐19533), and AKT inhibitor (HY‐10108) were purchased from MedChemExpress (MCE, 700874‐72‐2). Lactate, a‐cyano‐4‐hydroxycinna acid (CHC), and N‐acetyl‐L‐cysteine (NAC) were purchased from Sigma (Victoria, BC, Canada).

### 2.2 Cloning and DNA construction

To construct different length of CD38 promoters, fragments were amplified from H1299 cells' genomic DNA by PCR and were then cloned into pGL3‐Basic Vector (Promega, Madison, WI, USA) at the Hind III‐/Kpn I sites. The primers are listed in Table 1.

**TABLE 1:** Primers used for PCR amplifications.

Gene	Genebank accession number	Primer (5’>3′)
CD38	NM_001775.4	Forward: CGGGGTACCAGCCATTAT
		CCTCAGCAAACTAACAC
		Reverse: CCCAAGCTTTACATCCTT
		ATACTCCCTCCGCTAAC
CD38	NM_001775.4	Forward: CGGGGTACCTATTATTAC
		TGTGTGCCAGACCCTGC
		Reverse: CCCAAGCTTTACATCCTT
		ATACTCCCTCCGCTAAC
CD38	NM_001775.4	Forward: CGGGGTACCGATTACCAT
		GTGCCAGGTATTGTGCT
		Reverse: CCCAAGCTTTACATCCTT
		ATACTCCCTCCGCTAAC

### 2.3 Western blot analysis

The cells were washed with PBS, and then the cells were scraped off to prepare cell lysate by adding 1 × sample buffer. Protein extract after various treatments was electrophoresed and transferred to nitrocellulose (NC) membrane (GE Healthcare, Piscataway, NJ, USA). The extraction and isolation of membranous and cytoplasmic protein were performed according to the instructions for the Membranous and Cytoplasmic Protein Extraction Kit (Beyotime Institute of Biotechnology, Shanghai, China). The NC membrane was blocked in 5% nonfat milk at room temperature for 1 h and then probed with the primary antibodies overnight at 4°C. Except Anti‐β‐actin was diluted with 5% skim milk at a ratio of “1:5000″, the remaining antibodies were all diluted with 5% skim milk at a ratio of “1:1000”. The NC membranes were extensively washed three times, and then incubated with anti‐mouse (no. 7076) or anti‐rabbit (no. 7074) horseradish peroxidase‐conjugated secondary anti‐body (Cell Signaling Technology). Following removal of the secondary antibody, the membranes were scanned by FluorChem FC3 (ProteinSimple, San Jose, CA, USA).

### 2.4 Quantitative real‐time RT–PCR analysis

Cells were treated as indicated and total mRNA was isolated using TRIzol, according to the manufacturer's protocols. The obtained RNA was transcribed using PrimeScript First Strand cDNA Synthesis Kit (TaKaRa‐Bio, DaLian, China). The cDNA was mixed with ABI‐SYBR Green Master Mix (Applied Biosystems, Carlsbad, CA, USA), and the mixture was subjected to amplification using an ABI 7500 Real‐time PCR System (Applied Biosystems). The primers are listed in Table 2. Each sample was repeated in triplicate and analyzed using the Relative Quantification Software (Applied Biosystems).

**TABLE 2:** Primers used for RT‐PCR amplifications.

Gene	Genebank accession number	Primer (5’>3′)
CD38	NM_001775.4	Forward:AGACTGCCAAAGTGTATGGGAT
		Reverse: GGAAGTGTTGAATTCACCACAC
β‐actin	NM_001101.5	Forward: CCAACCGCGAGAAGATGAC
		Reverse: GAGGCGTACAGGGATAGCACA

### 2.5 Wound healing assay

Wound healing assay cells were seeded in a 6‐well plate at a concentration of 1 × 10^6^ cells/well and allowed to form a confluent monolayer for 24h. Then the transfected monolayer cells were scratched with 200 μL pipette tips, washed with PBS to remove floating cells, and photographed by a phase‐contrast microscope at 100× magnification (Olympus, Shinjuku‐ku, Tokyo, Japan) (time 0). Cells were further incubated with DMEM for 24 h or 48 h and photographed again (at 24 h and 48 h). The numbers of cells migrated to 0 h wound area were counted.

### 2.6 Dual luciferase reporter assays

Various reporter constructs were co‐transfected with Renilla luciferase vector into the cells. After 48 h, cells were lysed and activities of firefly luciferase and Renilla luciferase were analyzed, following the manufacturer's instruction. Each experiment was repeated in triplicate using a multimode microplate reader (TriStar LB941; Berthold Technologies, Bad Wildbad, Germany). The results are expressed as mean of triplicates ± SD.

### 2.7 Cell invasion assays

For the assessment of cell motility, the CHEMICON cell invasion assay was performed in an Invasion Chamber (Millipore, Billerica, MA, USA). Cells were seeded in triplicate at a density of 3 × 10^5^ cells/chamber. After 48 h, cells that had not moved to the lower wells were removed from the upper face of the filters using cotton swabs, and cells that had moved to the lower surface of the filter were stained by using a Cell Invasion Assay Kit. (CHEMICON, No. ECM550). Cell migration was quantified by visual counting after being photographed by a phase‐contrast microscope at 100x magnifications (Olympus, Shinjuku‐ku, Tokyo, Japan). Experiments were performed in triplicate. Mean values for three random fields were obtained for each well.

### 2.8 Short interfering RNA transfection

The cells (50%–60%) were transfected with 10 nM of short interfering RNA (siRNA) using 1 μL of GenMute siRNA Transfection Reagent (SignaGen Laboratories) in 6‐well plates. All the siRNAs were purchased from RiboBio Company (Guangzhou, China). After transfection for 48 h, cells were deprived of serum and growth factors for 12 h and then treated with lactate (Roche, San Francisco, CA, USA) for 3 h and harvested. The sequences of the siRNAs are listed in Table 3.

**TABLE 3:** Sequences of siRNA.

Gene	Genebank accession number	Target sequence (5′‐3)
siCD38	NM_001775.4	UUUGGCAGUCUACAUGUCUCAUCUC
siSnail	NM_005985.4	CAAATACTGCAACAAGGAA
siCD73	NM_002526.4	GAACCUGGCUGCUGUAUUG
siTEAD1	NM_021961.6	GCCCUGUUUCUAAUUGUGGTT

### 2.9 Cell cAMP assays

Cell cAMP assay was performed according to the manufacturer's instructions. Cells were seeded onto 96‐well plates at a cell density of 4000 cells per well. After transfection with CD38 cDNA for 48 h, the cell were lysed and incubated with cAMP‐AP conjugate and anti‐cAMP antibody in the second antibody‐coated plate for cAMP quantitation (cAMP‐Screen Direct System; T1505, Applied Biosystems, Foster City, CA 94404 USA). The absorbance of the samples was measured at wavelength of 492 nm using a microplate reader. There should be an inverse correlation between signal intensity (light units) and intracellular cAMP concentration.

### 2.10 NAD/NADH assays

Cells were harvested, and NAD+/NADH content was measured by using the NAD+/NADH Assay Kit with WST‐8 (Beyotime Institute of Biotechnology, Shanghai, China), according to instructions.

### 2.11 Statistical analysis

Statistical analysis was performed using Student's *t*‐test for the comparison of two groups or one‐way analysis of variance for the comparison of more than two groups followed by Tukey's multiple comparison test. For multiple testing, a Bonferroni post hoc test of *p* values was done. Statistical calculations were performed using GraphPad Prism (GraphPad, San Diego, CA, USA). Data were expressed as means ± SD of at least three independent experiments. A *p* value < 0.05 was considered statistically significant.

## 3 RESULTS

### 3.1 CD38 induces the Epithelial–Mesenchymal Transition program in lung cancer cells

**FIGURE 1:**
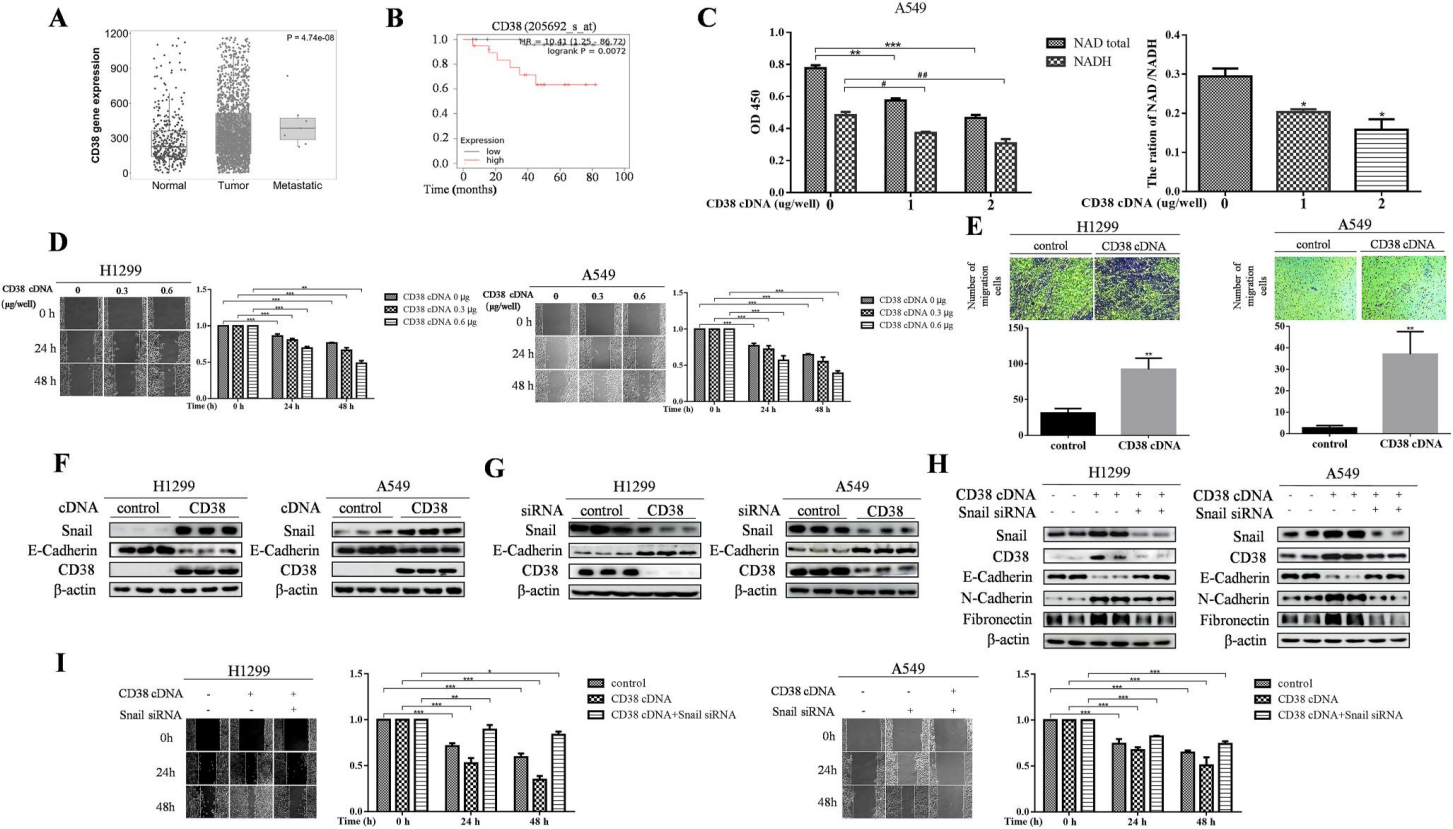
CD38 induces the Epithelial–Mesenchymal Transition (EMT) program in lung cancer cells (A) Public data TNM plot database was used to analysis of the significant expression of CD38 in lung normal tissues, lung adenocarcinoma, and metastatic lung adenocarcinoma. (B) Kaplan–Meier Plotter database was selected to analyze the prognosis of CD38 in lung adenocarcinoma. (C) A549 cells was treated with CD38 cDNA for 48 h. Intracellular levels of NAD were measured using the NAD/NADH Quantification Kit. (*p < 0.05, **p < 0.01, ***p < 0.001, ^#^ *p* < 0.05, ^##^ *p* < 0.01, for difference from untreated control by ANOVA with Dunnett's correction for multiple comparisons.) (D) Wound Healing Assay and (E) Cell Invasion Assays revealed an increase in migration as well as invasiveness in the cells overexpressing CD38 (upper panel). **p < 0.01, ***p < 0.001 for difference from control by ANOVA with Dunnett's correction for multiple comparisons. (F, G), Western blot shows Snail and E‐Cadherin expression in cells after transfection with CD38 cDNA or CD38 siRNA. *β*‐actin was a loading control. (H) At day 2, following co‐transfection with CD38 cDNA and Snail siRNA, Western blot analysis was conducted on A549 and H1299 cells to assess the expression levels of Snail, N‐Cadherin, E‐Cadherin, Fibronectin, and CD38. *β*‐actin was utilized as a loading control. (I) The assessment of cell migration in A549 and H1299 cells following co‐transfection of CD38 cDNA and Snail siRNA was conducted using the Wound healing assay. The quantification was present in right panels. *p < 0.01, **p < 0.01, and ***p < 0.001 for difference from control by ANOVA with Dunnett's correction for multiple comparisons.

In order to understand the role of CD38 in lung cancer, protein expression was first analyzed by using the publicly available data from lung cancer patient cohorts (https://tnmplot.com/analysis/). Compared to normal tissues, CD38 is highly expressed in primary human lung tumor tissues and metastatic lung cancer cells (Figure 1A). Moreover, increased CD38 expression was found to positively correlate with shorter overall survival of lung patients (https://kmplot.com/analysis/) (Figure 1B). To explore the potential mechanism by which CD38 regulates lung cancer development, we performed migration and invasion assays to examine its ability to induce EMT in CD38‐overexpressed NSCLC cells. Consistent with its reported NADase activity, the cells showed dose‐dependently decreased total NAD^+^ levels when CD38 is ectopically introduced into A549 cells (Figure 1C). Interestingly, CD38 overexpression led to an increased motility and invasiveness in wound closure and Matrigel‐coated Boyden chamber assays (Figures 1D and E). Western blot analyses confirmed that E‐cadherin expression is largely inhibited in CD38‐overexpressed A549 and H1299 cells (Figure 1F), Notably, this occurred with a parallel increase in EMT‐inducing transcription factor Snail protein levels. Conversely, depletion of CD38 caused robust increase and decrease in E‐cadherin and Snail expression, respectively (Figure 1G). Since Snail plays a critical role in inducing EMT by repressing E‐cadherin expression, we reasoned that CD38 might regulate Snail expression to activate cancer cell EMT program. To test this hypothesis, we silence Snail expression in CD38‐overexpressed cells. Overexpression of CD38 enhanced the accumulation of Snail protein in association with the suppression of E‐cadherin levels, as well as induction of fibronectin and N‐cadherin (Figure 1H). Remarkably, the induction of EMT program by CD38 overexpression was fully reversed after knockdown of Snail protein. A similar result was obtained in the cell motility assay (Figure 1I). Together, these data suggested that Snail is an essential mediator of the CD38‐inducing EMT process.

### 3.2 CD38/A2AR axis regulates Snail‐dependent lung cancer cell Epithelial–Mesenchymal Transition

**FIGURE 2:**
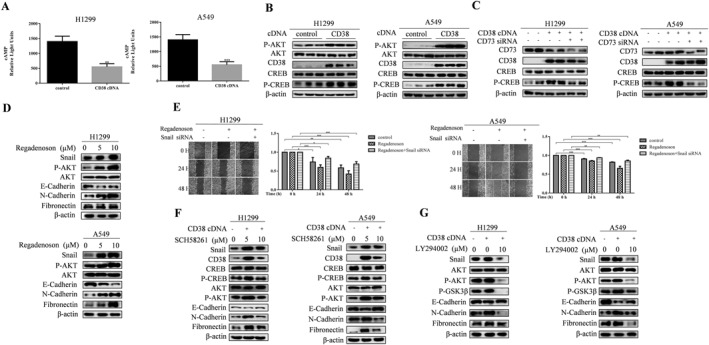
CD38/A2AR axis regulates Snail‐dependent lung cancer cell Epithelial–Mesenchymal Transition (EMT). (A) cAMP levels were measured by competitive immunoassay using cAMP‐Screen Direct System kit after treatment with transfection of CD38 cDNA. This assay provides an inverse correlation between light units and intracellular cAMP concentration, indicating that an increase in cAMP levels in CD38‐overexpressed cells. The bars represent the mean ± S.D. of triplicates. **p < 0.01, ***p < 0.001 for difference from control by ANOVA with Dunnett's correction for multiple comparisons. (B) Western blot analysis of P‐AKT, AKT, CD38, P‐CREB, and CREB in indicated cells at 48h after infection with CD38 cDNA. *β*‐actin was a loading control. (C) At day 2, following co‐transfection with CD38 cDNA and CD73 siRNA, Western blot analysis was conducted on A549 and H1299 cells to assess the expression levels of P‐CREB, CREB, CD73, and CD38. *β*‐actin was utilized as a loading control. (D) H1299 and A549 cells were treated with 0, 5 and 10 μM Regadenoson, and Western blot was conducted 6h later to determine the levels of Snail, P‐AKT, AKT, N‐Cadherin, E‐Cadherin, and Fibronectin. *β*‐actin was a loading control. (E) Wound Healing Assay show that the cell migration was undermined by treated with Regadenoson and Transfection of Snail siRNA. The quantification was present in the right panel. *p < 0.05, **p < 0.01, ***p < 0.001 for difference from control by ANOVA with Dunnett's correction for multiple comparisons. (F) A549 and H1299 cells were transfected with CD38 cDNA for 24 h and further treated with 10 μM SCH58261 for 6 h. Western blot was performed to examine the expression of Snail, CD38, P‐CREB, CREB, AKT, P‐AKT, N‐Cadherin, E‐Cadherin, and Fibronectin. *β*‐actin was a loading control. (G) A549 and H1299 cells were transfected with CD38 cDNA for 24 h and further treated with 10 μM LY294002 for 6 h. Western blot was performed to examine the expression of Snail, P‐AKT, AKT, N‐Cadherin, E‐Cadherin, and Fibronectin. *β*‐actin was a loading control.

The primary function of CD38 is to degrade the NAD+, the above results raise questions on how CD38 regulates the Snail expression. It was recently reported that the enzymatic functions of CD38 as part of an CD38/CD203a/CD73 complex that mediates the sequential conversion of NAD + to adenosine.^9^, ^17^ Adenosine binds to the Gαs‐coupled receptor A2AR to trigger the cAMP/PKA/CREB and PI3K/AKT pathways. Indeed, we observed an increase in cAMP levels in CD38‐overexpressed A549 and H1299 cells (Figure 2A). Western blot for phosphorylated AKT (p‐AKT) and phosphorylated‐CREB (p‐CREB), the direct indictors of activation of AKT and PKA pathways, showed a robust increase in levels of p‐AKT and p‐CREB after the ectopic expression of CD38 (Figure 2B). Furthermore, the silencing of CD73 fully suppressed CD38‐induced p‐CREB (Figure 2C), suggesting that adenosine might be a key downstream mediator of CD38 signaling. To determine the functional relevance of adenosine/A2AR in CD38‐induced EMT, we first treated the cells with Regadenoson, a pharmacological agonist of A2AR. Interestingly, Regadenoson stimulation efficiently caused the increased expression of the mesenchymal markers, N‐cadherin, and fibronectin, coupled with decreased epithelial marker E‐cadherin (Figure 2D), this occurred with a parallel increase of p‐AKT and Snail levels. Markedly, the ability of Regadenoson‐induced cellular motility was blunted by the knockdown of Snail protein in H1299 cells (Figure 2E), implying that A2AR is functionally relevant for Snail‐inducing EMT. To establish conclusively whether A2AR mediates induction of EMT program induced by CD38, we treated cells with an antagonist of A2AR and SCH58261 in CD38‐overexpressed cells. As expected, Snail, N‐cadherin, and fibronectin protein levels increase, while E‐cadherin levels are suppressed, and the levels of p‐AKT and p‐CREB are induced in a CD38‐sensitivity fashion in both A549 and H1299 cells (Figure 2F). Remarkably, the promoting effect of CD38 on EMT program is fully ablated by the treatment of SCH58261. It is well established that activation of AKT pathway has been associated with the acquisition of aggressive phenotype, AKT phosphorylates GSK‐3β, and inhibits its activity, inducing Snail protein stability and nuclear translocation, which subsequently promotes EMT.^18^ In fact, treatment of cells with the AKT inhibitor, LY294002 diminished GSK‐3β phosphorylation, and reversed the expression of Snail and the EMT markers exerted by overexpression of CD38 (Figure 2G). Taken together, the data indicate that a CD38/A2AR axis regulates Snail‐dependent lung cancer cell EMT through the AKT/GSK‐3β pathway.

### 3.3 Lactate‐induced ROS Is Associated with CD38 expression

**FIGURE 3:**
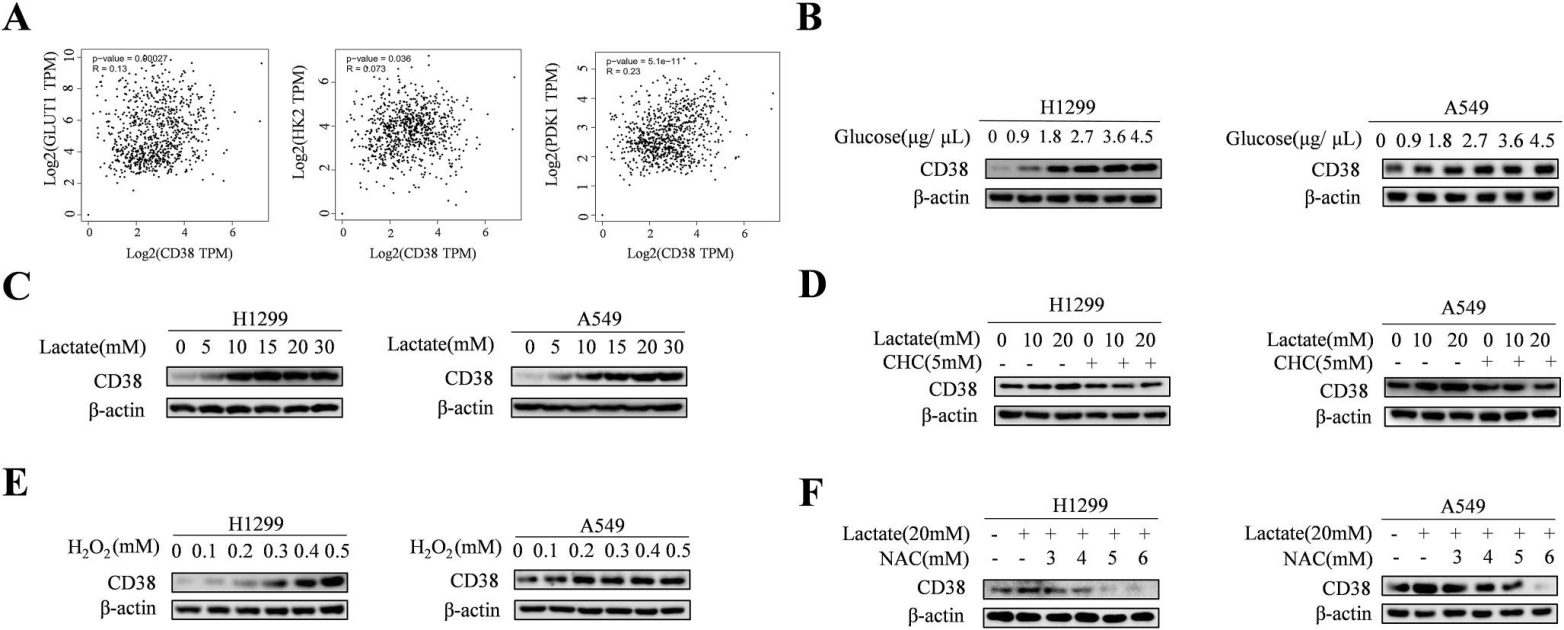
Lactate‐induced ROS is associated with CD38 expression (A) In the GEPIA2 dataset analysis of CD38 with glycolysis markers, results show the positive correlations between CD38 and glycolysis markers. (B) H1299 and A549 cells were treated with indicated concentrations of glucose for 3 h, and the expression levels of CD38 were analyzed by Western blot in NSCLC cells. *β*‐actin was a loading control. (C) H1299 and A549 cells were treated with indicated concentrations of lactate for 3 h, and expression levels of CD38 were analyzed by Western blot in NSCLC cells. *β*‐actin was a loading control. (D) H1299 and A549 cells were treated with 0, 10, and 20 mM of lactate in the presence or absence of CHC. Western blot was used to determine CD38 protein level. *β*‐actin was a loading control. (E) H1299 and A549 cells were treated with indicated concentrations of H_2_O_2_ for 3 h, and expression levels of CD38 were analyzed by Western blot in NSCLC cells. *β*‐actin was a loading control. (F) H1299 and A549 cells were treated with NAC in the indicated concentrations for 4 h and treated with lactate (20 mM) for 3 h. Western blot examines CD38 protein level.

CD38 is highly expressed in lung and cervical cancer in contrast with its low expression in prostate cancer.^6^, ^7^, ^8^ The above results indicating the involvement of CD38 in the regulation of Snail‐dependent EMT, prompt us to investigate how CD38 is regulated in lung cancer cells. Given that CD38 is intimately linked with cancer metabolism, we considered the possibility that CD38 activity might induce adaptation of cellular metabolism, which in turn modulate the expression of CD38. Since NSCLC is highly glycolytic, to test this hypothesis, we first examine the relationship of CD38 with the expression of genes related to glycolysis such as glucose transporter 1 (Glut1), Hexokinase 2 (HK2) and pyruvate dehydrogenase kinase‐1 (PDK‐1) by analyzing gene expression data from GEPIA2 (http://gepia2.cancer‐pku.cn/#index) database. Notably, a significant positive correlation between the expression of CD38 and all of these genes was revealed (Figure 3A), suggesting the potential link of cancer cell metabolism reprogramming with CD38 expression. We therefore sought to determine whether the metabolic player could modulate CD38 expression. We first evaluated whether modulation of glycolysis influence CD38 expression. Markedly, dose‐dependent glucose stimulation increased the expression of CD38 (Figure 3B). There is an accumulating evidence indicating that glycolysis‐derived lactate functions as signaling molecule and plays an important role in metastasis and poor prognosis. Therefore, we asked whether lactate might also have an important role in regulation of CD38. Importantly, exogenous lactate triggered a dose‐dependent increase in CD38 expression (Figure 3C). The cancer cells can use exogeneous lactate to fuel the tricarboxylic acid cycle (TCA) and oxidative phosphorylation (OXPHOS) and monocarboxylate transporter 1 (MCT1) is a key transporter for the uptake of lactate into cells;^19^ therefore, we used MCT1 inhibitor a‐cyano‐4‐hydroxycinnamate (CHC) to block lactate transport. As shown in Figure 3D, levels of CD38 induced by lactate was decreased in the presence of CHC, supporting the transport of lactate into cells for the regulation of CD38 expression. Our previous work has demonstrated that reactive oxygen species (ROS) generated by lactate‐fueled OXPHOS activates Hippo pathway,^14^ prompting to test whether the role of ROS is functionally relevant for CD38 regulation. To examine this, we treated lung cancer cells with H_2_O_2_ to mimic the effect of oxidative stress. Markedly, oxidative stress from exposure to H_2_O_2_ stimulated dose‐dependent increase in the levels of CD38 (Figure 3E). Furthermore, we monitored the lactate‐induced CD38 expression in the presence of anti‐oxidant N‐acetyl‐L‐cysteine (NAC). The levels of CD38 induced by lactate was attenuated significantly by NAC in both A549 and H1299 cells (Figure 3F), providing strong evidence for the role of lactate‐induced ROS in the regulation of CD38.

### 3.4 Activation of TEAD by lactate increases CD38 expression

**FIGURE 4:**
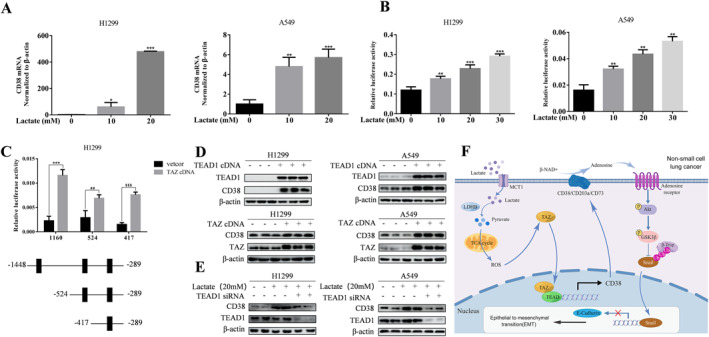
Activation of TEAD by lactate increases CD38 expression (A) CD38 mRNA was measured by qRT‐PCR in cells with indicated the doses of lactate treatment. Values represent the relative reduction of CD38 mRNA normalized to *β*‐actin. The bars represent the mean ± S.D. of triplicates. *p < 0.05, **p < 0.01, ***p < 0.001 for the difference from the control cells by ANOVA with Dunnett's correction for multiple comparisons. (B) A549 and H1299 cells were co‐transfected with CD38 promoter and controlled Renilla luciferase reporter gene plasmid and treated with indicated lactate concentrations after 48 h. Luciferase activity was determined and normalized using the dual luciferase reporter system. **p < 0.01, ***p < 0.001, for difference from control cells by ANOVA with Dunnett's correction for multiple comparisons. (C) Schematic representation of three different lengths of the CD38 promoter normal construction. Cells were transiently co‐transfected with TAZ cDNA and three different lengths of the CD38 promoter luciferase reporter, and luciferase activity was determined. The bars represent the mean ± S.D. of triplicates. ***p < 0.001, ^#^ *p* < 0.05, ^$$$^ *p* < 0.001, for difference from control cells by ANOVA with Dunnett's correction for multiple comparisons. (D) Western blot analysis of CD38, TEAD1, or TAZ at 48h after infection with TEAD1 cDNA or TAZ cDNA in indicated cells. *β*‐actin was a loading control. (E) Transfection of A549 and H1299 cells with TEAD1 siRNA or their respective controls for 48 h; cells were treated with the indicated concentrations of lactate (20 mM) for an additional 3 h. Levels of CD38 and TEAD1 were detected by western blot. *β*‐actin was a loading control.

We next sought to explore the molecular mechanism by which lactate regulates CD38 expression. We first conducted quantitative real‐time PCR to measure the mRNA levels of CD38 gene. qRT‐PCR results indicated that dose‐dependent lactate increased CD38 mRNA levels in both A549 and H1299 cells (Figure 4A). Furthermore, a luciferase reporter harboring CD38 promoter was generated. Strikingly, the promoter activity of CD38 was significantly induce by lactate (Figure 4B), indicating that lactate transcriptionally regulates CD38 expression. Our previous finding that lactate‐fueled OXPHOS increases activity of transcriptional co‐activator TAZ, a major downstream effector of Hippo pathway, raised the possibility of TAZ‐controlled CD38 expression. As TAZ functions as transcriptional co‐activator, the interaction of TAZ with transcriptional factor TEAD is essential for TAZ activity. Examination of the promoter sequence of CD38 showed three potential binding sites of TEAD (Figure 4C). Consistent with our hypothesis, forced expression of TEAD or TAZ led to markedly increase in CD38 expression (Figure 4D). Importantly, silencing of TEAD using siRNA significantly decreased lactate‐induced CD38 levels (Figure 4D). Furthermore, a set of CD38 deletion constructs containing various TEAD binding sites revealed that the first and last sites are critical in mediating lactate‐induced CD38 expression. Taken together, these data indicated that TAZ mediates lactate‐induced CD38 expression.

## 4 DISCUSSION

The molecular mechanisms underlying the high expression of CD38 and its promotion of tumor development in tumor cells have not been elucidated. Our current work shows that lactate, which is caused by tumor metabolism remodeling, upregulates the expression of CD38 through the Hippo‐TAZ pathway. The highly expressed CD38 converts NAD + to adenosine through the CD203a/CD73 complex, and adenosine binds and activates its receptor A2AR, inducing the expression of Snail and promoting the invasion and metastasis of lung cancer cells. This finding elucidates a new perspective on the role of organismal NAD + metabolism in tumor development.

Initially identified as a T cell activation marker, CD38 has now been confirmed to be a multifunctional protein, with its primary function being the breakdown of NAD+.^4^ It plays an important role in diseases such as infection and aging.^20^ Interestingly, the role of CD38 in tumor initiation and progression has provided some conflicting data. For example, in pancreatic cancer and prostate cancer, low CD38 expression increases tumor cell survival.^6^, ^21^ On the other hand, high expression of CD38 promotes cervical cancer cell proliferation, and CD38 significantly enhances tumor growth in nude mice.^7^ Cell cycle analysis reveals that CD38 induces cell accumulation in the S phase and inhibits apoptosis in cervical cancer cells. The different role of CD38 in tumors has not yet been elucidated. Consistent with our findings, Moss laboratory utilized the CRISPR/Cas9 gene knockout of CD38 in A549 lung adenocarcinoma cells, which inhibited cell growth, cell invasion, and xenograft tumor growth in nude mice.^8^ Overall, our data supports the role of CD38 in the development of lung tumors in humans, and anti‐CD38 therapy may have therapeutic potential in lung cancer.

Our results confirm that ADPR/cADPR catalyzed by CD38 can be further converted into adenosine by CD203 and CD73 enzymes. Adenosine binding and activation of its receptor A2AR induce the expression of Snail, thereby promoting the invasion and metastasis of lung cancer cells. CD38 is a type II transmembrane enzyme with its catalytic activity directed toward the extracellular space.^4^ How this specific orientation of CD38 on the cell membrane could regulate intracellular levels of NAD+ is still controversial. Our results could link this particular structure of CD38 to its potential biological roles in tumor development. Furthermore, the underlying molecular mechanisms of the adenosine‐A2AR signaling pathway in lung cancer invasion and progression is not well‐defined. Our results confirm that adenosine activates the downstream AKT signaling pathway through A2AR, promoting the expression of Snail and the process of epithelial–mesenchymal transition (EMT).

Previous studies have documented that AKT/GSK‐3β/Snail pathway regulates the migration and invasion of various types of cancer cells.^22^, ^23^, ^24^ In this pathway, AKT phosphorylates GSK3β at the Ser9 site, leading to a decreased activity of GSK3β. GSK3β binds to and phosphorylates the Snail transcriptional repressor, causing Snail mRNA to be transported to the cytoplasm and degraded.^18^ Our results showed that the AKT inhibitor, LY294002, suppressed GSK‐3β phosphorylation in parallel with the expression of Snail and the EMT markers induced by overexpression of CD38, indicating the crucial role of AKT/GSK‐3β/Snail in CD38/A2AR signaling.

As a result of metabolic reprogramming, tumor cells rely on aerobic glycolysis and secrete large amounts of lactate into the tumor microenvironment. High concentrations of lactate in the microenvironment are associated with poor clinical prognosis.^25^ Our laboratory and other studies have shown that lactate plays an important role as a signaling molecule in tumor development.^16^, ^26^, ^27^, ^28^ We previously found that the accumulation of lactate and the interaction with oxidative metabolism induce the activation of TAZ.^14^ The induction of TAZ involves the MCT1‐mediated transport of lactate into the mitochondria. Mitochondrial ROS, generated by lactate‐driven OXPHOS, stimulate AKT activity, leading to the inhibition of the GSK‐3β/β‐TrCP ubiquitination pathway and subsequent accumulation and activation of DNMT1. DNMT1 methylation of the LATS2 gene promoter overcomes the inhibition of TAZ activity. This work further demonstrates that lactate‐induced TAZ promotes the expression of CD38. Taken together, this study describes that the lactate‐induced CD38 enhances our understanding of the dynamic relationship between metabolism and tumor development. In addition, rigorous dissection of interplay between CD38 and lactate signal pathway may offer potential therapeutic benefits for therapy.

## AUTHOR CONTRIBUTIONS

Yating Lu, Yang, Tao Chang, Qiyun Jiang, Chenfeng Yang and Chunzhe Fu performed the in vitro experiments. Zhihao Wu, Yuanpeng He and Huijun Wei coordinated in all experiments and done supervision. Zhihao Wu designed this study and the experiments and wrote the manuscript. All authors read and approved the final manuscript.

## CONFLICT OF INTEREST STATEMENT

The authors have no conflict of interest.

## ETHICS STATEMENT

Not applicable.

## Data Availability

The data used or analyzed in this study are available from the corresponding author upon reasonable request.
